# Epidemiological and molecular analysis of circulating fowl adenoviruses and emerging of serotypes 1, 3, and 8b in Egypt

**DOI:** 10.1016/j.heliyon.2021.e08366

**Published:** 2021-11-15

**Authors:** Amany Adel, Ahmed Abd Elhalem Mohamed, Mahmoud Samir, Naglaa M. Hagag, Ahmed Erfan, Mahmoud Said, Abd El Satar Arafa, Wafaa M.M. Hassan, Mohamed E. El Zowalaty, Momtaz A. Shahien

**Affiliations:** a Reference Laboratory for Veterinary Quality Control on Poultry Production, Animal Health Research Institute, Agriculture Research Center, Giza 12618, Egypt; b Zoonosis Science Center, Department of Medical Biochemistry and Microbiology, Uppsala University, Uppsala, SE 75 123, Sweden

**Keywords:** Fowl adenovirus, Loop1, Hypervariable, Adenoviruses, Includion body Hepatitis, Epidemiology, Sequence, Molecular analysis, Emerging

## Abstract

Fowl adenoviruses (FAdVs) are a large group of viruses of different serotypes. They are responsible for inclusion body hepatitis, adenoviral gizzard erosion, and hepatitis hydropericardium syndrome. The present study presents a comprehensive overview of FAdVs in Egypt, with a focus on the epidemiological features of virus serotypes across the country. We conducted molecular investigation of multiple FAdV species based on the genetic signature of hypervariable regions 1–4 in the loop1 (L1) region of the *hexon* gene. Epidemiologically, the Nile Delta governorates showed high positivity of FAdVs, which were more commonly found in broilers than in layers. Genetically, species D and serotype 8a/E dominated, and the findings also revealed the emergence of new FAdV serotypes 1, 3, and 8b. The comparative analysis of hypervariable regions in the L1 region of the *hexon* gene revealed variables specific to each virus serotype. In silico predictions of L1 region revealed variations in the molecular structure and predicted the antigenic epitopes which may affect the cross-antigenicity between the different FAdV species and serotypes.

## Teaser

Fowl adenovirus, Loop1, HVRs, Egypt.

## 1 Introduction

Fowl adenoviruses (FAdVs) are non-enveloped icosahedral viruses containing linear dsDNA genome and belong to the genus *Aviadenovirus* in the family *Adenoviridae* (Harrach et al., 2019). Based on their genome sequence, members of the *Adenoviridae* family are classified into five genera: *Mastadenovirus*, *Atadenovirus*, *Siadenovirus*, *Aviadenovirus*, and *Ichtadenovirus* (Berk, 2007; Fitzgerald et al., 2020). FAdVs are avian viruses which infect primarily young brolier chicks and generally show no clear clinical symptoms and may be isolated from apparently healthy birds (Adair and McFerran, 2008). Infected birds may experience various clinical signs such as respiratory problems, reduced egg production, arthritis, tenosynovitis, uneven growth, or enteritis with variable degrees of mortality (Adair and McFerran, 2008).

FAdVs are grouped into five species (FAdV-A through FAdV-E) based on restriction enzyme digestion patterns (Zsak and Kisary, 1984) and 12 serotypes (FAdV-1 to -8a and 8b to -11) were identified based on serum cross-neutralization tests (Hess, 2000). The genus *Siadenovirus* causes hemorrhagic enteritis in turkeys, marble spleen disease of pheasants, and avian adenovirus splenomegaly virus of chickens. In addition, the genus *Atadenovirus* includes the virus which causes egg drop syndrome (Hafez, 2011). The most common associated diseases caused by FAdVs of Group I in chickens are inclusion body hepatitis (IBH), hepatitis hydropericardium syndrome (HHS), and adenoviral gizzard erosion (AGE) (Domanska-Blicharz et al., 2011; Nakamura et al., 1999; Wells and Harrigan, 1974).

Highly virulent FAdV-4 (species C) appears to have a more serious role than others in the etiology of IBH and HHS and leads to high mortality rates ranging from 20% to 80% (Asthana et al., 2013). Some FAdV-1/A strains cause AGE which is associated with growth retardation (Lim et al., 2012; Thanasut et al., 2017; Zadravec et al., 2013). Although all the 12 FAdV serotypes have been associated with outbreaks of IBH (Hess, 2013), the most common strains belong to serotypes of species FAdV-D and FAdV-E and have been isolated in multiple countries (Schachner et al., 2016; Zadravec et al., 2013). IBH is most commonly detected in broilers chicks aged 2–20 weeks (Hafez, 2011; Şahindokuyucu et al., 2020). IBH is an emerging adenoviral disease causing detrimental economic consequences on the global poultry industry (Nakamura et al., 2011; Mittal et al., 2014; Schachner et al., 2016; Niczyporuk, 2017)

Histologically and pathologically, IBH is characterized by necrotic foci in a pale hemorrhagic liver which include basophilic intranuclear inclusion bodies (El-Deeb and Mandour, 2019; Dar et al., 2012). Lymphocytic infiltration, cellular necrosis, and degeneration of infected organs have also been observed within three to nine days postinfection, and atrophy of bursa and swelling in the kidney have been reported in some cases (Grafl et al., 2013; Matos et al., 2016; Steer et al., 2015). In general, IBH is not very serious and has low mortality rates ranging from 5% to 10%, although mortality can be up to 30% owing to secondary infections (Mase et al., 2012). Clinically, affected birds crouch and show ruffled feathers along with enteric symptoms such as diarrhea and death or recovery may occur within 48 h (Li et al., 2018).

FAdVs may be isolated from both healthy and sick chicks (Kaján et al., 2013; Niczyporuk, 2016), and their role as primary pathogens is not clear because their pathogenicity may vary among strains belonging to the same serotype (Absalón et al., 2017; Erny et al., 1991; Slaine et al., 2016). The mortality and severity of FAdV infections can be influenced by various factors, including chicken breed; for example, light Sussex chickens were found to have higher susceptibility to IBH than Rhode Island chickens (Schachner et al., 2018). In addition, the status of the birds’ immune system or concurrent infection with other immunosuppressive infectious agents is a significant factor (Toro et al., 2000).

FAdVs also possess potential immunosuppressive ability due to reducing humoral and cell-mediated immunity, which can increase the susceptibility of infected birds to other pathogens (Schonewille et al., 2008; Singh et al., 2006). Serotype 8, for example, was found to induce depletion in the lymphoid organs, including the bursa, spleen, and thymus, leading to a reduction in antibody production (Saifuddin and Wilks, 1992).

The hexon is the major protein of FAdVs, its molecule consists of two conserved basement regions (P1 and P2) and four loops (L1, L2, L3, and L4) in its outer portion, with the loops containing seven hypervariable regions (HVRs) (Crawford-Miksza and Schnurr, 1996). L1 has the highest number of variables for HVRs, and L1, L2, and L4 contain antigenic and immunogenic portions which are used in typing and differentiation while L3 has no antigenic impact (Niczyporuk, 2018).

In Egypt, FAdVs in the poultry population have drawn little interest, which has resulted in no vaccination measures have been developed against such viruses in the various poultry sectors. While there are limited studies about the current situation of FAdVs, there have been reports on the spread of the serotypes which induce IBH, including FAdV-8a (Radwan et al., 2019) and FAdVs 2/11, which belong to the FAdVs species D (El-Tholoth and Abou El-Azm, 2019; Elbestawy et al., 2020). The need to increase awareness about the consequences of FAdV infections in Egypt makes it essential to investigate their impact and evolution.

The current study therefore focused on the evolution of FAdVs, while also investigating possible serotypes that have not been previously detected in Egypt. The study aimed also to provide an updated epidemiological overview of these viruses in Egypt.

## 2 Materials and methods

### 2.1 Samples

A total of 340 samples were collected from different governorates in Egypt during 2019 and 2020. Samples included 302 cloacal swabs obtained from different poultry farms. Each sample represented pooled swabs collected from suspected chickens in each farm and samples were submitted to the Reference Laboratory for Veterinary Quality Control on Poultry Production (Giza, Egypt) for routine pre-slaughter screening for specific viruses. The reamining 38 samples were obtained from visceral organs (livers, spleen, and bursa) of morbid chicken flocks.

### 2.2 Epidemiological data analysis

Epidemiological data were processed and analyzed using Microsoft Excel spreadsheet (version 2010). The geographical distribution of positive cases in Egypt was mapped using Tableau software version 2020.1 (Tableau Software, LLC, Seattle, WA, USA) (https://www.tableau.com/).

### 2.3 Virus isolation and propagation in chicken embryo liver cells

Samples were tested for virus isolation using primary chicken embryo liver (CEL) cells to confirm the presence of live infectious viruses. Primary cell culture was prepared from liver embryos which were harvested aseptically from 13 to 15 day-old specific-pathogen-free embryonated chicken eggs (Koum Oshein, El-Fayoum, Egypt). Cell culture preparation and propagation of viruses were performed as was previously described (Mohamed Sohaimi et al., 2019).

### 2.4 Molecular detection of FAdVs

#### 2.4.1 Extraction of viral nucleic acid

Viral nucleic acid was extracted using the QIAamp MinElute Spin Kit (Qiagen, GmbH, Germany) according to the manufacturer's instructions as follows: a volume of 200 μL of the swab sample fluid or tissue homogenate supernatant was incubated with 200 μL of AL lysis buffer and 25 μL of Qiagen protease at 56 °C for 15 min. Thereafter, 250 μL of absolute ethanol was added to the lysate, which was then washed and centrifuged. Nucleic acid was finally eluted using 100 μL of elution buffer. DNA extracts were kept at -20°C for further analysis.

#### 2.4.2 Amplification of viral nucleic acid using conventional PCR

Conventional PCR was performed using in-house designed specific primers for the L1 region of the *hexon* gene of FAdVs. The specific oligonucleotide primers were used for the amplification of the L1 loop of the hexon gene of different adenovirus serotypes. The primers were synthesised by metabion (Munich, Germany). The nucleotide sequences of the primers were as follows: adeno-F- 5ʹ-ACATGGGAGCGACCTACTTCGACA-3ʹ and adeno-R- 5ʹ-TCGGCGAGCATGTACTGGTAAC-3ʹ. The expected product size was 700 bp. PCR amplification was accomplished using an EmeraldAmp Max PCR Master Mix (Takara, Japan) in a total volume of 25-μL consisting of 12.5 μL of EmeraldAmp Max PCR Master Mix, 1 μL of forward and reverse primers (working concentration 20 pmol), 5.5 μL of PCR-grade water, and 5 μL of extracted DNA. The reactions were run in Biometra T3000 thermal cycler as follows: denaturation step at 95 °C for 5 min; 35 cycles of secondary denaturation at 94 °C for 30 s, annealing at 60 °C for 45 s, and extension at 72 °C for 1 min; and a final extension step at 72 °C for 10 min.

#### 2.4.3 Sequencing of the L1 region of the hexon gene

The amplified PCR products of appropriate size were subsequently purified using a QIAquick Gel Extraction Kit (QIAGEN, Hilden, Germany). The purified PCR products were subjected to sequencing reactions using a Big Dye Terminator v3.1 Cycle Sequencing Kit (Applied Biosystems, Foster City, CA) according to the manufacturer's specifications, and the reaction product was purified by exclusion chromatography using a DyeEX 2.0 Spin Kit. The recovered materials were sequenced using a 3500 XL DNA Analyzer (Applied Biosystems, Foster City, USA).

#### 2.4.4 Sequence and phylogenetic analyses of the L1 region of the hexon gene

Multiple nucleotide sequence alignment was performed using BioEdit software version 7.0 using the ClustalW alignment algorithm and the percentage identity matrices between different virus sequences was determined. Neighbor-joining phylogenetic trees were constructed using the distance-based method in MEGA software version 11. The trees included the sequences generated in the current study and additional availabe sequences of strains of FAdV serotypes were downloaded from GenBank (https://www.ncbi.nlm.nih.gov/genbank/).

The L1 region of the hexon protein was modeled using the Expasy database, SWISS-MODEL (https://swissmodel.expasy.org/) (Arnold et al., 2006 and Waterhouse et al., 2018), and the three-dimensional structure of the simulated protein was visualized using the PyMOL 1.1 software (DeLano, 2002).

In silico prediction of antibody epitopes in the L1 region of different viruses representing the species of FAdVs in the present study were achieved using IEDB Analysis Resource (http://tools.iedb.org/main/) using a semi-empirical method as was previously reproted (Kolaskar and Tongaonkar, 1990). The predicted antigenic epitopes were colored yellow when they were above the threshold (values >1).

## 3 Results

### 3.1 Epidemiological analysis

Different types of samples were tested for FAdVs during 2019 and 2020 in the present study. As shown in Table 1, cloacal swabs collected from apparently healthy flocks were the most abundant type of sample. Such samples were routinely screended during pre-slaughter examination for specific viruses such as avian influenza virus H9N2, avian influenza H5 subtype virus, infectious bronchitis virus, and Newcastle disease virus. In addition, organs of suspected cases including livers, spleens, and bursa were examined for signs of the disease. These cases exhibited depression, loss of weight, and liver inflammation which appeared pale and enlarged. Some cases showed respiratory manifestations and abnormal gestures due to secondary infections with other pathogens (Table 2).

**Table 1:** Collective epidemiological data of the examined and positive cases in the present study.

Data	No. of examined samples	No. of positive cases (positivity %)
**Region and Location:**		
**Lower Egypt (the Nile Delta)**	213	16 (7.5%)
Menofia	98	6
Qalyoubia	91	2
Daqahlia	14	3
Beheira	2	1
Cairo	2	2
Gharbia	4	0
Sharqia	2	2
**Upper Egypt**	127	3 (2.4%)
El Wadi El Gedid	30	1
Asyut	31	1
El Fayoum	1	0
Giza	40	0
El Menia	24	0
Sohag	1	1
**Type of breed:**		
Balady	35	1 (2.9%)
Broiler	114	13 (11.4%)
Layers	34	3 (8.8%)
Unknown breed	157	2 (1.2%)
**Clinical signs:**		
Apparently healthy	302	7 (2.3%)
Clinically morbid	38	12 (31.6%)
**Total**	340	19

**Table 2:** Complete descriptive data of FAdV positive samples in the present study.

	Sample ID	GenBank Acc.no.	Serotype	Governorate	Age	Type of chicken	Clinical signs	Other pathogens
1	AD1–2019	MW699421	2/11 (species D)	Sharqia	67 d	Layers-Novogen	Depression Drop in production	Negative
2	AD2–2019	MW699422	2/11 (species D)	Qalyoubia	40 d	Broiler	No clinical signs	Negative
3	AD-3-2020	MW699423	2/11 (species D)	Sharqia	21 d	Broiler-Indian River	Loss of weight Depression Gastrointestinal disorders	Negative
4	AD4–2020	MW699425	2/11 (species D)	El Minya	grower	Broiler	No clinical signs	Negative
5	AD5–2020	MW699424	2/11 (species D)	Daqahlia	35 d	Broiler Cobb	Loss of weight Depression Gastrointestinal disorders	Negative
6	AD6–2020	MW699426	2/11 (species D)	Menofia	34 w	Broiler Indian River breeders	Loss of weight Depression Gastrointestinal disorders	not examined
7	AD7–2020	MW699427	2/11 (species D)	Menofia	10 d	Broiler Indian River	Loss of weight Depression Gastrointestinal disorders	not examined
8	AD8–2020	MW699428	2/11 (species D)	El Wadi El Gedid	grower	Broiler	No clinical signs	Negative
9	AD9–2020	MW699429	2/11 (species D)	Sohag	8 d	Broiler	Gastrointestinal disorders Loss of weight depression	Salmonellosis
10	AD10–2020	MW699430	2/11 (species D)	Daqahlia	unknown	Layers breeders	Gastrointestinal disorders Loss of weight Depression Abnormal gesture	Marek's disease virus
11	AD11–2019	MW712883	8a (species E)	Cairo	55 d	Layers	Depression and Ruffled feather Gastrointestinal disorders	H9
12	AD12–2019	MW712884	8a (species E)	Daqahlia	4 d	Cobb broilers	Dullness, Ruffled feather	Negative
13	AD13–2020	MW712885	8a (species E)	Qalyoubia	66 d	Balady	No clinical signs	Negative
14	AD14–2019	MW712886	8a (species E)	Cairo	37d	Broiler Hubbard	Weight loss Ruffled feather Abnormal gesture Gastrointestinal disorders	Reovirus
15	AD15–2020	MW712887	8b (species E)	Menofia	37 d	Broiler	No clinical signs	Negative
16	AD16–2020	MW712888	8b (species E)	unknown	unknown	Unknown	No clinical signs	not examined
17	AD17–2020	MW689188	1 (species A)	Beheira	33 d	Broiler	Gastrointestinal disorders Loss of weight Depression Respiratory manifestation Inflammation in bursa	Infectious bronchitis virus
18	AD18–2020	MW699419	3 (species B)	unknown	unknown	Unknown	unknown	not examined
19	AD19–2020	MW699420	3 (species B)	Menofia	25 d	Broiler	Gastrointestinal disorders Loss of weight depression	Coccidia

Chickens were the main target species in the present study and various breeds were examined, as shown in Table 1. Broiler breeds were abundant because the most examined cases were the pre-slaughter flocks, with 13 broiler flocks being found positive out of the 19 positive cases, as shown in Tables 1 and 2.

**Figure 1:**
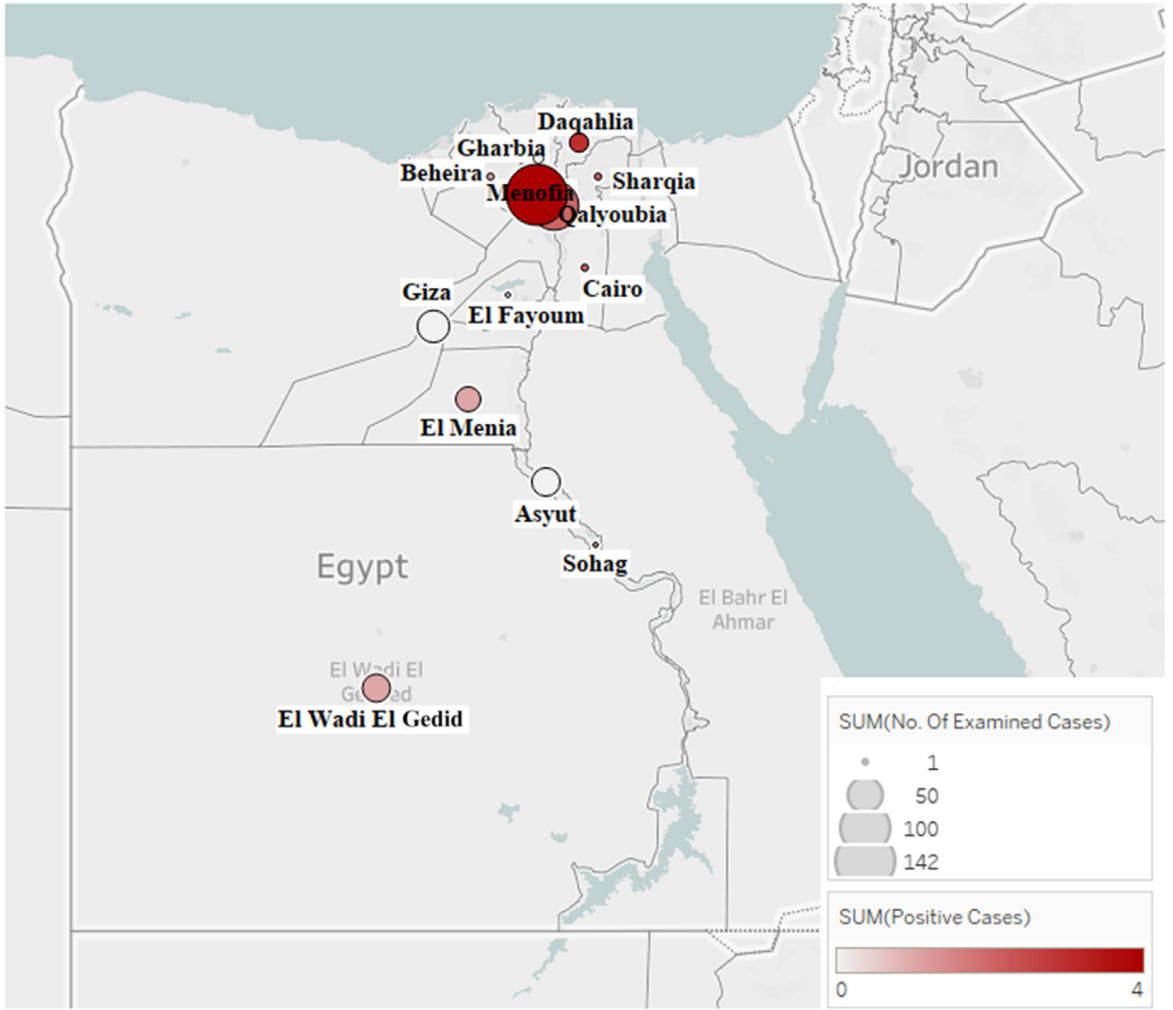
Geographic map of Egypt showing the locations of farms where the samples were collected and positive cases of FAdVs were reported in the present study. The density of examined cases is represented by the size of the circular mark at each governorate, while the number of positive cases is represented by the color intensity of the circular marks.

As shown in Table 1 and Figure 1, the collected samples were mainly obtained from Lower Egypt (the Nile Delta) governorates, with some samples were obtained from Upper Egypt governorates. The Nile Delta region therefore had a higher number of positive cases (7.5%) than Upper Egypt (2.4%), with Menofia and Daqahlia (in the Nile Delta) having the majority of positive cases. FAdVs were found to infect chicken at different ages, from younger than one week to 34-week-old (Table 2).

### 3.2 Virus isolation

**Figure 2:**
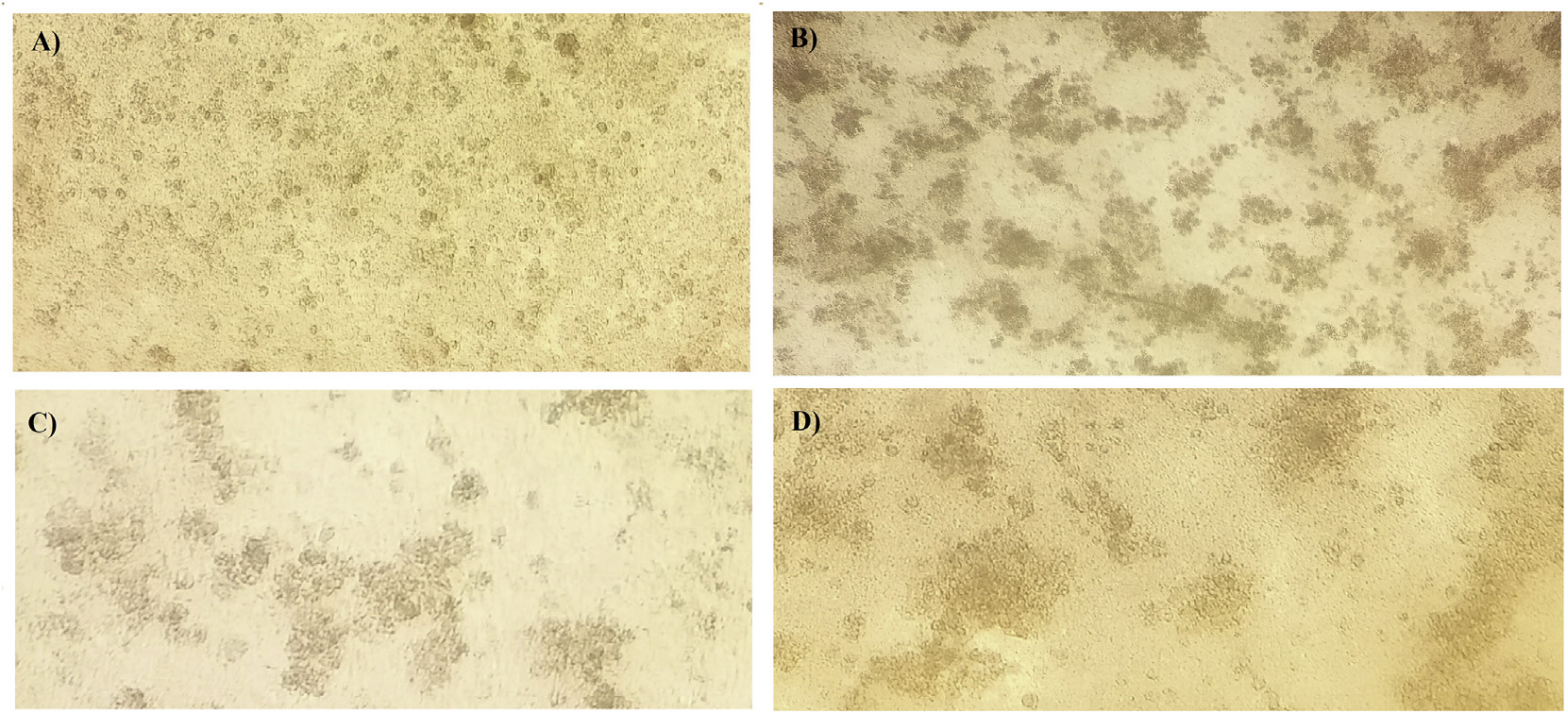
The cytopathic effect of different fowl adenovirus species on CEL cells after 36 h post infection. Infected cells show slaughing and clumping of detached cells. A) Negative control CEL cells B) CPE of CEL cells infected with FAdV- 8a/E C) CPE of CEL cells infected with FAdV-8b/E and D) CEL cells infected with FAdV-D showing less severe CPE than other species in the present study.

The positive samples AD1, AD12, and AD15, which represented serotypes 2/11, 8a and 8b, respectively, were propagated in primary CEL cells and cytopathic effects were observed including cell sloughing and clumping (Figure 2). The CPE effect of each FAdV species was variable and it was observed that species D virus had a slower cytopathic effect on CEL cells than other species when tested the same titer (10^7^ TCID50/100 μL).

### 3.3 PCR amplification of the *hexon* gene

Nineteen samples were positive for FAdV by conventional PCR. The L1 region of the *hexon* gene was the target in the present study, and the amplified fragment had a molecular weight of approximately 700 bp.

### 3.4 Sequencing of L1 of *hexon* gene

The L1 region of the *hexon* gene was sequenced and the nucleotide sequence of each product was deposited to GenBank under the accession numbers as shown in Table 2. Using BLAST (https://blast.ncbi.nlm.nih.gov/Blast.cgi), 10 out of the 19 virus sequences generated in the present study had 99% sequence identity with strains isolated in 2019–2020 (accession numbers MT127412.1 and MT759842) which belonged to FAdV species D. Moreover, four isolates were similar to serotype 8a viruses, such as strain TR59 (accession number KT862810). In addition, two isolates showed high similarity with viruses of serotype 8b, such as strain 764 (accession number KT862811). Two isolates had 99% sequence similarity with species B viruses, such as the M/2015 Debrecen strain (accession number MG953201) and serotype 3 strains, such as ATCC VR-828 (accession number AF339916). One isolate showed 98% identity with the CELO reference strain of species A (accession number U46933). The 19 sequences generated in the present study were aligned with other reference sequences whcih represented the five FAdV species (A–E). Based on the alignment results, the pairwise identity matrix and phylogenetic analyses were accomplished.

### 3.5 Phylogenetic analysis of L1 of the *hexon* gene

**Figure 3:**
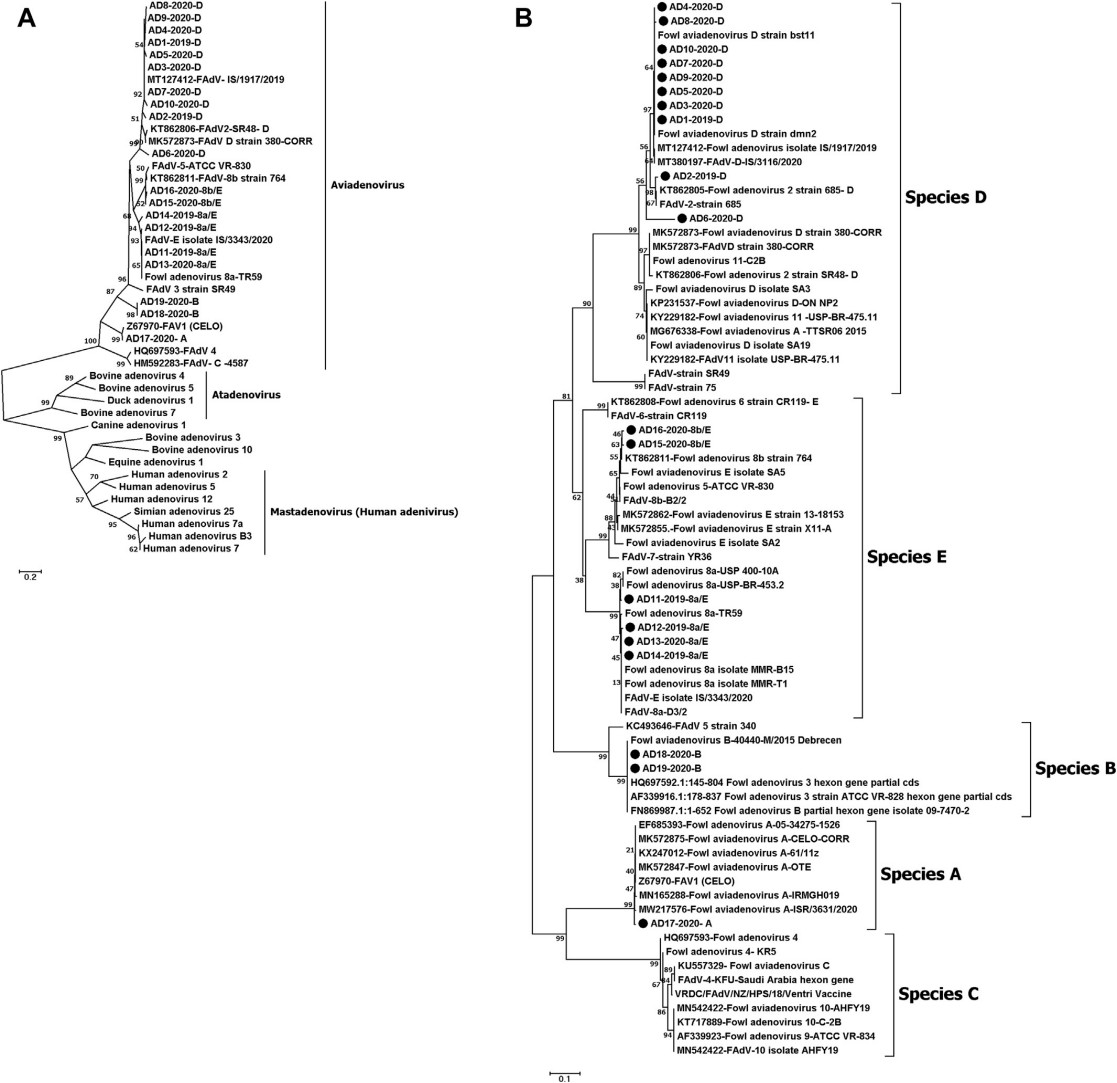
Phylogenetic analysis of nucleotide sequences of Loop1 (L1) region on hexon gene of fowl adenovirus. A) The phylogenetic tree shows that the 19 sequences generated in the present study belong genetically to the genus *Aviadenoviruse*. B) the phylogenetic tree of aviadenoviruses in Egypt shows the 10 strains genetically clustered into species D (AD1-AD10), 6 strains belong to species E (AD11-AD16), 2 strains clustered into species B (AD18, AD19) and only one strain belongs to species A (AD17). The trees were generated using MEGA software version 11 with neighbor-joining using the distance-based methods with bootstrap of 1000 replicates.

Based on genetic variations in L1, target sequences generated in the current study were genetically classified as follows: one isolate was assigned to serotype 1, two isolates were assigned to serotype 3/B, ten isolates belonged to serotypes of species D, and six isolates belonged to species E. The latter were closely related to each other but were divided into two serotypes, with four isolates were assigned to serotype 8a and two to 8b of species E (Figure 3).

### 3.6 Pairwise identity matrix of nucleotide and amino acid sequences

**Figure 4:**
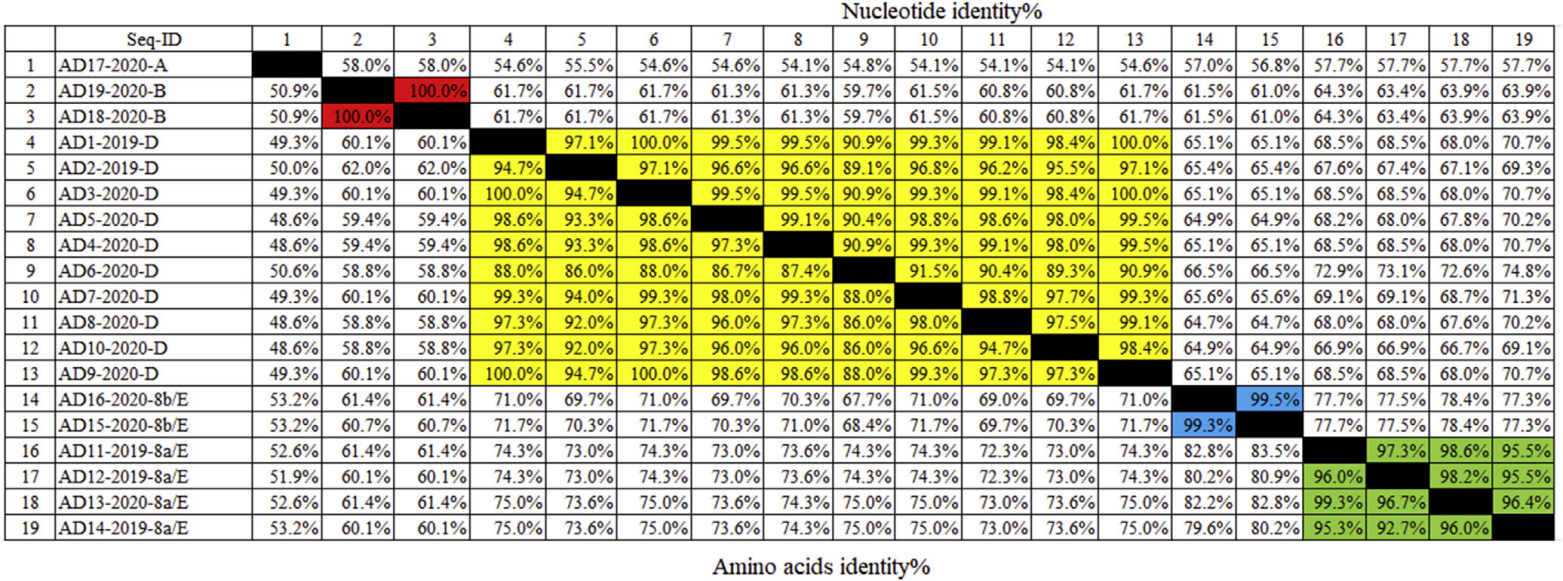
Pairwise analysis of nucleotide and amino acid sequences of the 19 fowl adenovirus strains in the present study and other related isolates based on partial sequencing of L1 region of the *hexon* gene.

The pairwise identity matrix (Figure 4) revealed high similarity among the samples of each species. Briefly, the identities between the 10 isolates of species D was found within a range of 98% to 100%, and these isolates were very similar to the Egyptian strain dmn2-2019 (accession number MH782424) and the strain IS/3116/2020 (accession number MT380197). However, AD6-2020-D showed low similarity with other viruses of species D, including the Egyptian viruses, with identities of 88% to 91%. The four isolates assigned to species E/8a showed 95.5% to 99% identity and were similar to the Egyptian strain MMR-T1-2019 (accession number KT781517) and the strain IS/3343/2020 (accession number MT759841). Despite their genetic relatedness, the isolates of serotype 8a/E and 8 b/E had low similarity, which ranged from 77.3% to 82.5%.

### 3.7 Mutation analysis of amino acid residues

**Figure 5:**
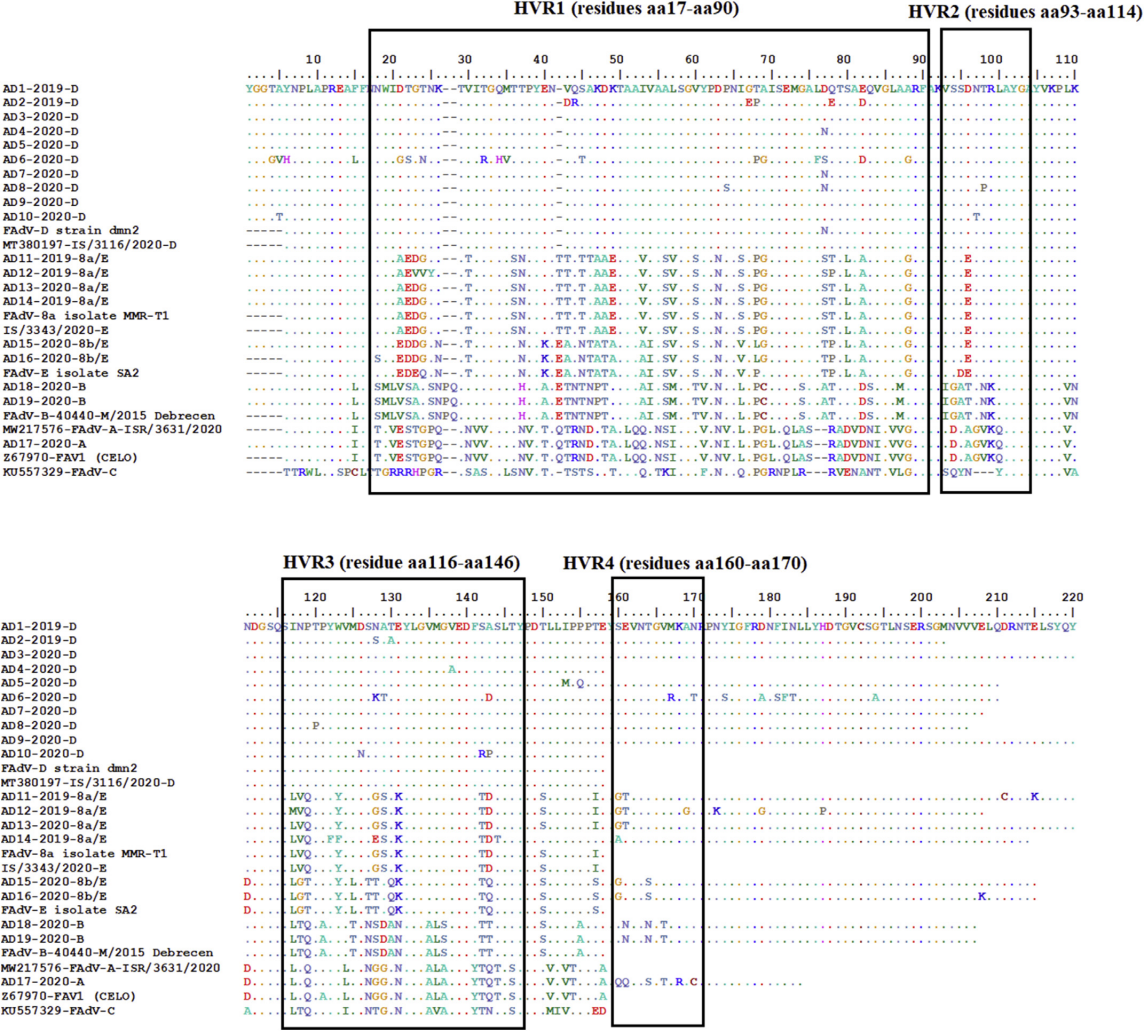
Amino acid sequences alignment of Loop1 (L1) region of the *hexon* gene of different fowl adenovirus species in the current study; the alignment shows the 4 hypervariable regions (HVRs1-4) according to Niczyporuk (2018). Numbering of amino acid residues was based on the consensus of different FAdV species alignment.

The amino acid sequences were aligned (Figure 5) and analysis revealed variations in the four HVRs of the L1 region, with the numbering of the amino acid residues being based on the consensus of the different FAdV species alignment. As shown in Figure 5, isolates belonging to each species had the same residues in each region. However, AD12–2019, one of the isolates of serotype 8a/E, had a variation in three amino acid residues in HVR1 (^23^VVY^25^) and a substitution in the same region as T78P which were similar to those substitutions in isolates of serotype 8 b/E. The isolates of serotypes 8a, 3, and 1 shared the amino acid residue Q119 in HVR3. Likewise, AD6–2020 (which belongs to species D) had some drifts in its amino acid sequence, particularly ^67^PG^68^ and G88, compared to other isolates in the same group. These residues were similar to those in the isolates of serotype 8a and 1 in HVR1, and there were also unique mutations at D21G, T22S, T24N, T32R, Q34H, M35R, S45T, L76F, D77S, Q82D, and A88G, which were similar to those in viruses of species A and E.

**Figure 6:**
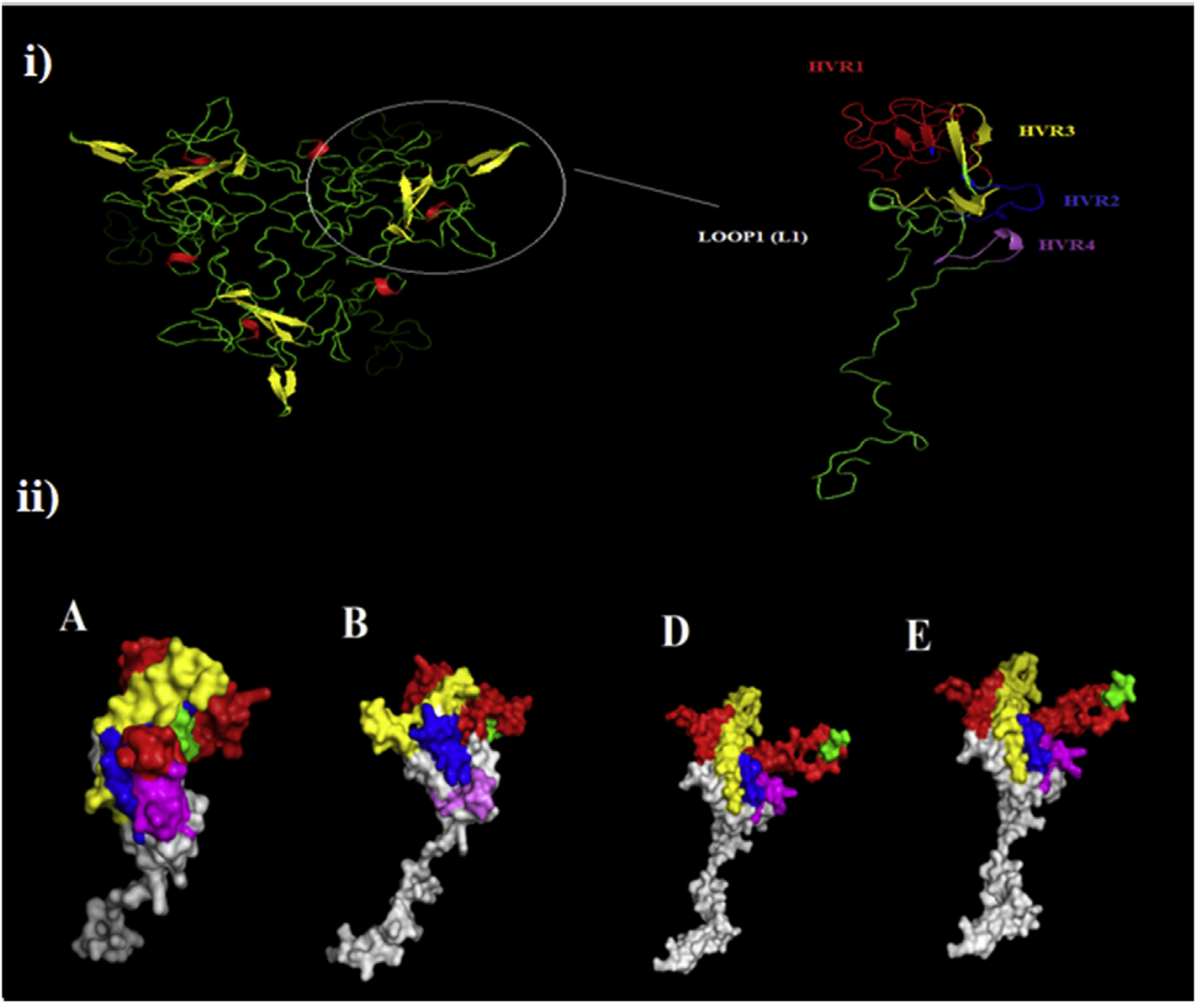
Modeling of L1 region structure of hexon protein. i) upper view of the tertiary structure of the hexon protein and lateral view of L1 region illustrates four HVRs. ii) lateral view of the molecular structure of the L1 region of viruses represent species A, B, D, and E; the comparison between these molecules shows variability in the structure of HVR1-4 at the top of the L1 region. Red: HVR1, Blue: HVR2, Yellow: HVR3, Purble: HVR4, Green: conservative species-specific sequence.

The proposed model for the L1 region was created based on the hexon protein of the avian adenovirus CELO strain (template no. 2iny.1.A). The created model illustrates the location of the four HVRs on the surface of the hexon protein as shown in Figure 6. The three-dimensional models of the different species in this study clarify the dissimilarities in structures of HVR1–4 of each serotype.

### 3.8 Prediction of linear antibody epitopes in the L1 region

The in silico prediction of antibody epitope peptides in the L1 region showed differences in the predicted linear epitopes among the FAdV species. As illustrated in Table 3 and Supplementary Figure 1, each FAdV species had a different number of predicted epitopes. The FAdV-1 virus showed the indicated deviations in its predicted epitopes compared with the other serotypes. In contrast, the viruses of serotypes of species B, D, and E shared some epitopes in HVR3 and HVR4. Although both FAdV- 8a and 8b were genetically related to species E, they did not share the same epitopes, particularly in HVR1 and HVR3. Moreover, AD6–2020, a FAdV-2/11 of species D showed more variations in some predicted antigenic peptides than other viruses of species D.

**Table 3:** Predicted antigenic peptides in the Loop1 region on the hexon gene in different fowl adenovirus species.

Species	No. of predicted peptides	Peptide	Species	No. of predicted peptides	Peptide
A	7	^27^TNVVGQM^33^	E/8a	7	^48^TAAVVASVSGS^58^
		^49^LQQVNSISGVVPNI^62^			^75^STTLAAQVGLA^85^
		^67^GLSQLA^72^			^99^AYGAYVKPL^107^
		^79^NIGVVGRF^86^			^113^QSLVQTPYYVMD^124^
		^94^VKQAYGAYVKPV^105^			^142^SLTYPDSLLIPPPI^155^
		^129^LGALAVED^136^			^180^INLLYHDTGVCSGT^193^
		^140^TLSYPDTVLVTPPTAYQQV^158^			^201^MNVVVEL^207^
B	8	^17^NSMLVSAT^24^	E/8b	8	^48^TAAAIASVSGS^58^
		^27^PQTVITG^33^			^75^TPTLAAQVGLAGRFAKV^91^
		^66^LGPCISEMS^74^			^99^AYGAYVKPL^107^
		^81^ADSVGLM^87^			^117^TTPYYVLDT^125^
		^101^AYGAYVKPV^109^			^128^QKYLGVM^134^
		^116^SLTQTAYW^123^			^142^SLTYPDSLLIPPPS^155^
		^133^LGALSVE^139^			^180^INLLYHDTGVCSGT^193^
		^144^SLTYPDSLLIPAPT^157^			^201^MNVVVEL^207^
		^182^INLLYHDTGVCSGT^195^			
D	5	^48^AAIVAALSGVYPD^60^	AD6-serotype D	5	^48^AAIVAALSGVYPD^60^
		^78^AEQVGLAARFAKV^90^			^78^ADQVGLA^84^
		^98^AYGAYVKPL^106^			^98^ AYGAYVKPL^106^
		^137^DFSASLTYPDTLLMP^151^			^141^SLTYPDTLLIPPPT^154^
		^179^INLLYHDTGVCSGT^192^			^179^FTLLYHDTGVCSAT^182^

## 4 Discussion

Recently, FAdVs have received increasing attention in the poultry industry globally, with limited studies were conducted in Egypt. FAdVs are classified into 12 serotypes that are divided into five species (A–E) (Hess, 2000), with only two serotypes (D and 8a/E) have been reported in Egypt (El-Tholoth and Abou El-Azm, 2019; Elbestawy et al., 2020; Radwan et al., 2019). To the authors knowldege, this is the first study to report the detection of FAdV- 8b, 1, and 3 in Egypt.

The current study aimed to determine the current epidemiological picture of FAdV in Egypt and investigated the possible geographical distribution of FAdVs in the country. Governorates in Lower Egypt have a large poultry sector which accounted for the majority of the examined and positive samples collected from this region in the present study, particularly Menofia, Qalyoubia and Daqahlia (Tables 1 and 2, Figure 1).

While virus infection usually leads to clinical signs in live chicks, FAdVs in the present study were detected in apparently healthy birds with no clinical signs, other than the birds were dull and depressed. The positivity of these healthy flocks could be related to the latency of FAdV infection, which causes silent infection until birds become stressed (El-Tholoth and Abou El-Azm, 2019; Fadly et al., 1980). Interestingly, we found birds of wide range of ages were infected with different FAdV serotypes. It was previously found that the most susceptible age for FAdV infection was reproted to be from two to 20 weeks (Hafez, 2011; Şahindokuyucu et al., 2020), however, in the present study viruses from three chicks of age less than two weeks old were isolated as shown in Table 2. Such early-age infections may be explained due to the vertical transmission from parental flocks (Grafl et al., 2013; Schachner et al., 2018).

In the present study, FAdV- positive sample (AD10) was also positive for Marek's disease virus (MDV). The two viruses were isolated from a layer flock in Daqahlia, where the birds suffered from loss of body weight, accompanied by depression with ruffled feathers and a drop in egg production. In addition, sample AD14 collected from a 37-day-old broiler flock was positive for reovirus, and the birds suffered from acute loss of body weight, malabsorption, and ruffled feathered, similar to a recent study which reproted the isolation of FAdVs from a poltry flock co-infected with avian reoviruses (Niczyporuk et al., 2021). Co-infection of FAdV and immunosuppressive pathogens was previously reported to increase morbidity and pathogenicity (McFerran and Smyth, 2000).

FAdV-1 of species A is the causative agent of AGE disease (Hess, 2000; Lim et al., 2012; McFerran and Smyth, 2000). FAdV was isolated from a unique case in the present study (sample AD17) which was collected from a 33-day-old broiler flock exhibiting gastrointestinal disorders. The chickens presented with a loss in body weight as well as respiratory signs and inflammation of the bursa. In addition to FAdV-1, this flock showed positivity for infectious bronchitis virus. These findings were typical form of AGE as was reported in previous outbreaks (Abe et al., 2001; Goodwin et al., 1993; Ono et al., 2001). Although, serotype 1/A is the main causative agent of AGE, sporadic cases were reported with serotypes 8a and 8b (Mase and Nakamura, 2014; Okuda et al., 2004). Isolation of FAdV-1 in the current study from sample (AD17) obtained from the bursa of Fabricius, along with the degenerative changes, supported the previous findings which indicated the immunosuppressive effect of FAdV-1 in infected broilers (Singh et al., 2006).

Additionally, strain AD11 which was isolated from a layer flock in Cairo was also positive for low-pathogenicity avian influenza A/H9N2 virus, and this flock showed symptoms of gasping, dullness, and gastrointestinal disorders. Furthermore, AD9 and AD19 were isolated from broilers with gastrointestinal disorders and were also positive for salmonella and coccidia infections, respectively. These results therefore support the immunosuppressive effect of FAdVs and their ability to exacerbate the severity of other diseases, despite vaccination and treatment (Niu et al., 2017; Radwan et al., 2019; Shivachandra et al., 2003).

Molecular detection tests of FAdVs in the current study were designed to differentiate between the types of FAdVs which may be circulating undetected in Egypt. The designed primers targeting the L1 region which is the most hypervariable region on the *hexon* gene and can be used to differentiate between the species of FAdVs (Hess, 2000; Niczyporuk, 2018).

In the current study, six FAdV serotypes were detected including previously detected serotypes of species D (FAdVs-2/11) and species E (FAdV-8a) (El-Tholoth and Abou El-Azm, 2019; Elbestawy et al., 2020; Radwan et al., 2019). FAdV species D was dominant in the present study, with 10 viruses (52.6% of positive samples) similar to FAdVs- 2/11 were detected as was previously reproted (Niczyporuk, 2018). Additionally, six viruses represented species E were detected and were classified as four viruses related to FAdV-8a (21.1%) and two viruses (10.5%) related to FAdV-8b. Two more viruses (10.5%) related to FAdV-3 were detected, and only one FAdV-1 was recorded. Although FAdV-4, the pathogenic type of FAdV, was not detected in the present study, a recent study reported the first detection of FAdV-4 in Egypt (Sultan et al., 2021), which was isolated from a cubb broiler flock at age of 32 days in Alexandria. The flock had a 15% mortality rate, and the examined post mortem lesions seemed typical of a pathogenic type of FAdV. The pathognomonic lesions were flappy heart with pericarditis and inflammatory fluid accumulation, in addition to an enlarged pale liver with petechial hemorrhage and necrotic foci (Sultan et al., 2021).

The L1 region is one of the four loop areas (L1, L2, L3, and L4) which contain immunogenic HVRs on the surface of the *hexon* gene (Crawford-Miksza and Schnurr, 1996). Moreover, the L1 region is considered a type/species-specific area (Niczyporuk, 2018; Raue et al., 2005), as it consists of four HVRs, with characteristic amino acid sequence lengths and compositions for each FAdV species (Niczyporuk, 2018). In addition, the HVRs of the L1 region load antigenic determinants specific for each serotype, particularly the HVR1 (Moffatt et al., 2000; Niczyporuk, 2018; Pichla-Gollon et al., 2007).

A comparable alignment of different amino acid sequences of species of FAdVs and other adenoviruses from different origins (human, canine, bovine) is shown in Supplementary Figure 2. The sequenced region is equivalent to the HVR of the first viral jelly-roll area (V1) which includes an extended loop (DE1) Rux et al. (2003).

Alignment of the L1 area of different serotypes of FAdVs in the present study showed dramatic deviations between species. Type-specific conservative amino acid residues occur in the peak of the L1 region at the end of HVR1 as was previously reported (Niczyporuk, 2018). Briefly, the alignment of FAdV strains in the present study as shown in Figure 5 revealed ^33^GQMTN^37^ in FAdV-1 and 8b. FAdV-3 and 8a possessed ^33^GQMTH^37^ and ^33^GQMSN^37^, respectively, although the amino acid sequence ^33^GQMTT^37^ was specific for FAdV-2/11/D. This conserved area is surrounded by the HVRs, and it has therefore been speculated to play a critical role in the immunogenicity and antigenicity of the virus (Singh et al., 2015). Thus, the surface structure must be variable in each species. The Egyptian FAdV-2/11 of species D revealed some mutations in both the AD2–2019 and AD6–2020 isolates. In particular, AD6–2020 showed unique substitutions which were not recorded in Egyptian FAdVs of species D strains and non-Egyptian viruses. The most important substitutions were found in the species-specific conserved area, which shifted from ^33^GQMTT^37^ to ^33^GHVTT^37^.

The comparative in silico simulation of the molecular structure of the L1 area in each FAdV species showed high dissimilarity in the structure of HVRs across species Figure 6. The prediction of antigenic epitopes also clarified the dramatic variations in the predicted epitopes between each species (Supplementary Figure 1), particularly those of FAdV-1. However, it appeared to have shared epitopes in the intermediate conserved areas between species D and E at 100–115 amino acid residues. Furthermore, there is another conserved peptide epitope in all the species located at amino acid residues 180–200. This means that each species may have characteristic-specific immunogenic properties. Accordingly, these predictions are comparable to those of previous studies which emphasized the lack of cross-antigenicity and protection between different FAdVs species (Niczyporuk, 2018; Schachner et al., 2018). While knowledge on epitope and antigenic mapping of fowl adenoviruses is lacking, studies on other types of adenoviruses reported that the neutralizing epitopes of each virus type are significantly variable, even if the viruses of different types are genetically related (Adam et al., 1998; Moffatt et al., 2000; Pichla-Gollon et al., 2007; Rux et al., 2003). Another remarkable finding was the variations in some predicted linear epitopes in the Egyptian isolate AD6–2020 as compared to those of species D (Supplementary Figure 1), which may indicate an important effect of intra-species antigenicity.

The possibility of inter-species recombination was previously reported (Das et al., 2017; Schachner et al., 2019; Singh et al., 2013). Such chimeric viruses could possess a wide range of cross-antigenic determinants, with additionally unique patterns of pathogenicity (Das et al., 2017; Schachner et al., 2019). It is therefore highly recommended to do complete genome sequencing of FAdVs circulating in Egypt to obtain a clear understanding of their genetic determinants, particularly as some viruses in AD6–2020 were found to share amino acid residues with other FAdV species.

## 5 Conclusions

FAdVs are among the important viruses which may affect the poultry industry both directly and indirectly. In the past few years, studies have highlighted the clinical cases, pathogenicity, and types of FAdVs in Egypt. The emergence of FAdVs-1,3, and 8b serotypes were reproted in the present study, in addition to the previously identified FAdV-2/11 of species D and FAdV-8a of species E. Furthermore, genetic comparative analysis of the HVRs in the L1 area of the *hexon* gene showed unique genetic deviations in two new strains belonging to species D (AD2–2019 and AD6–2020). In addition, the predicted antigenic epitopes showed inter- and intra-species variations. Further studies are required to determine the pathogenicity and establish the genetic and antigenic constitutions of different FAdV species in Egypt.

## Declarations

### Author contribution statement

Amany Adel: Conceptualization, Methodology, Formal analysis, Investigation, Writing - Original draft, Writing - review & editing, Data Curation, Visualization, and Supervision. Ahmed Abd ElHalem, Mahmoud Samir, and Mahmoud Said: Methodology, Investigation and Writing - review & editing. Naglaa M. Hagag, Ahmed Erfan: Resources, Investigation, and Writing - review & editing. Abd El Satar Arafa, Wafaa M. Hassan, and Momtaz A. Shahien: Project administration, Writing - review & editing, Supervision. Mohamed Ezzat El Zowalaty: Visualization, Validation, Analysis, Supervision, Writing-Original draft, and Writing - review & editing.

### Funding statement

The study was supported by funding from the Animal Health Research Institute. Open access funding was organized and enabled by Uppsala University, Sweden.

### Data availability statement

Additional data supporting the study findings are available in the supplementary material. Sequence data generated in the present study and supporting the conclusions of this article were deposited in the GenBank, National Library of Medicine, (NCBI) under accession numbers MW689188, MW699419, MW699420, MW699421, MW699422, MW699423, MW699424, MW699425, MW699426, MW699427, MW699428, MW699429, MW699430, MW712883, MW712884, MW712885, MW712886, MW712887, and MW712888.

### Declaration of interests statement

The authors declare no conflict of interest.

### Additional information

No additional information is available for this paper.
