# Supplementary material for: Epidemiological and molecular analysis of circulating fowl adenoviruses and emerging of serotypes 1, 3, and 8b in Egypt
Source: Heliyon. 2021 Nov 15;7(12):e08366. doi: 10.1016/j.heliyon.2021.e08366 (PMC8683735; doi:10.1016/j.heliyon.2021.e08366)
Supplement: Supplementary file 1 [file mmc1.pdf]

Supplementary file 1

Epidemiological and Molecular Analysis of Circulating Fowl Adenoviruses and Emerging of Serotypes 1, 3, and 8b in Egypt

Amany Adel,[1] Ahmed Abd Elhalem Mohamed,[1] Mahmoud Samir,[1] Naglaa M. Hagag,[1] Ahmed Erfan,[1] Mahmoud Said,[1] Abd El Satar Arafa,[1] Wafaa Hassan,[1] Mohamed E. El Zowalaty,[2] Momtaz A. Shahien [1]

[1] Reference laboratory for veterinary quality control on poultry production, Animal Health Research Institute, Agriculture Research Center, Giza 12618, Egypt

[2] Zoonosis Science Center, Department of Medical Microbiology and Biochemistry, Uppsala University, Uppsala, Sweden

Corresponding authors: (MEZ) elzow005@gmail.com and (AA) a.adel18784@gmail.com

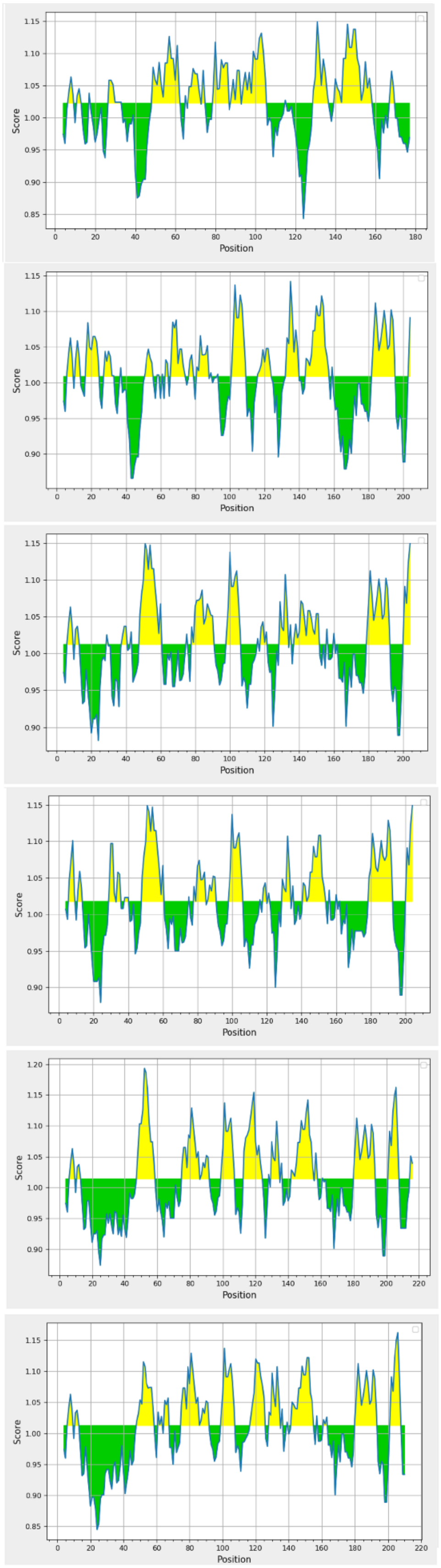

A

B

D

AD6-2020
Egypt/D

E/8a

E/8b

Comparative analysis of the predicted antigenic linear epitopes in the HVRs of the Loop1 region. In silico prediction of B cell epitope was conducted using Antibody Epitope Prediction server (IEDB Analysis Resource) using a semi-empirical method of Kolaskar and Tongaonkar,1990.
