# Supplementary material for: Epidemiological and molecular analysis of circulating fowl adenoviruses and emerging of serotypes 1, 3, and 8b in Egypt
Source: Heliyon. 2021 Nov 15;7(12):e08366. doi: 10.1016/j.heliyon.2021.e08366 (PMC8683735; doi:10.1016/j.heliyon.2021.e08366)
Supplement: Supplementary file 2 [file mmc2.pdf]

Supplementary file 2

Epidemiological and Molecular Analysis of Circulating Fowl Adenoviruses and Emerging of Serotypes 1, 3, and 8b in Egypt

Amany Adel,[1] Ahmed Abd Elhalem Mohamed,[1] Mahmoud Samir,[1] Naglaa M. Hagag,[1] Ahmed Erfan,[1] Mahmoud Said,[1] Abd El-Satar Arafa,[1] Wafaa Hassan,[1] Mohamed E. El Zowalaty,[2] Momtaz A. Shahien [1]

[1] Reference laboratory for veterinary quality control on poultry production, Animal Health Research Institute, Agriculture Research Center, Giza 12618, Egypt

[2] Zoonosis Science Center, Department of Medical Microbiology and Biochemistry, Uppsala University, Uppsala, Sweden

Corresponding authors: (MEZ) elzow005@gmail.com and (AA) a.adel18784@gmail.com

Comparative alignment of Loop1 region of different adenoviruses including fowl, human, bovine and canine adenoviruses.

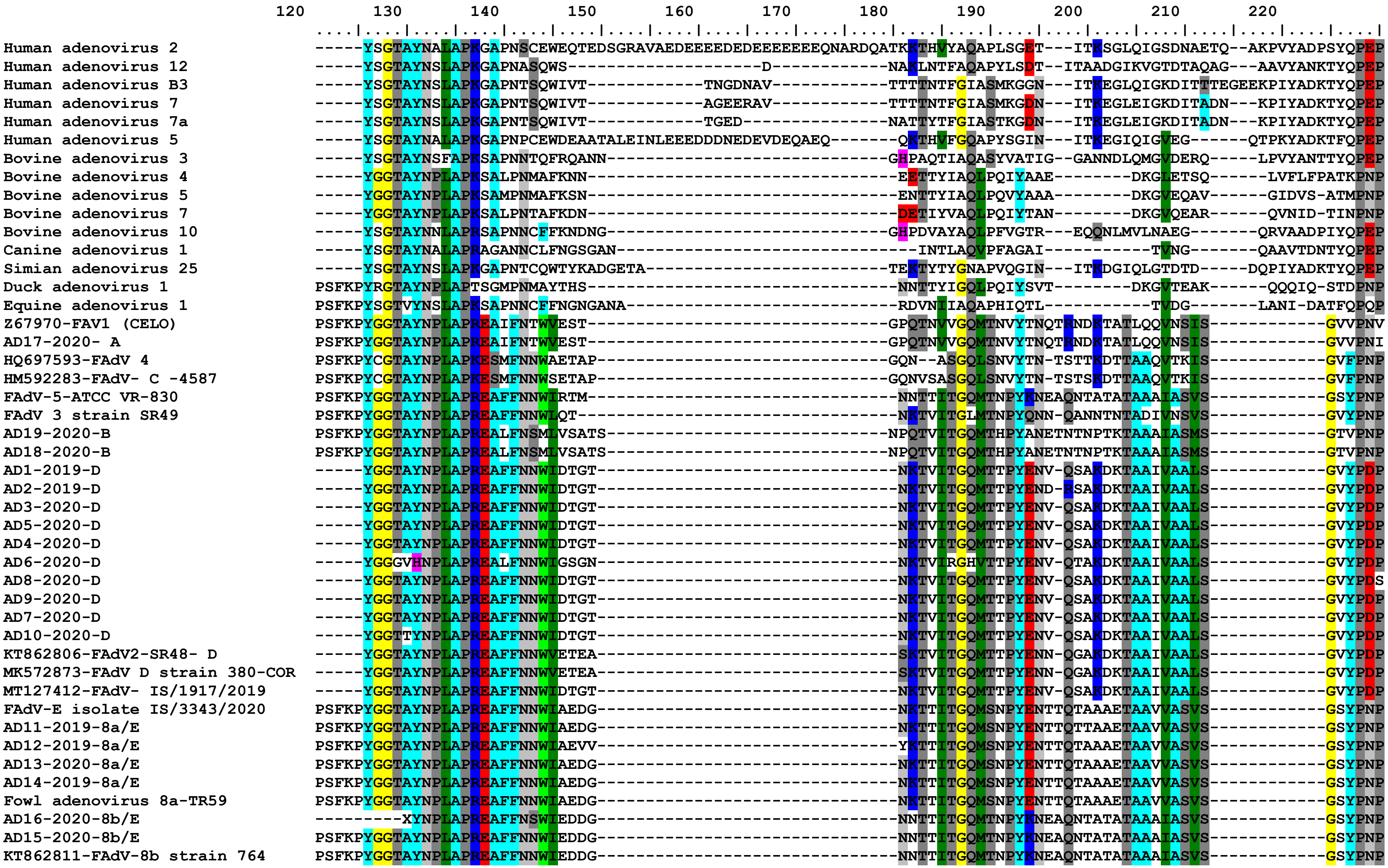

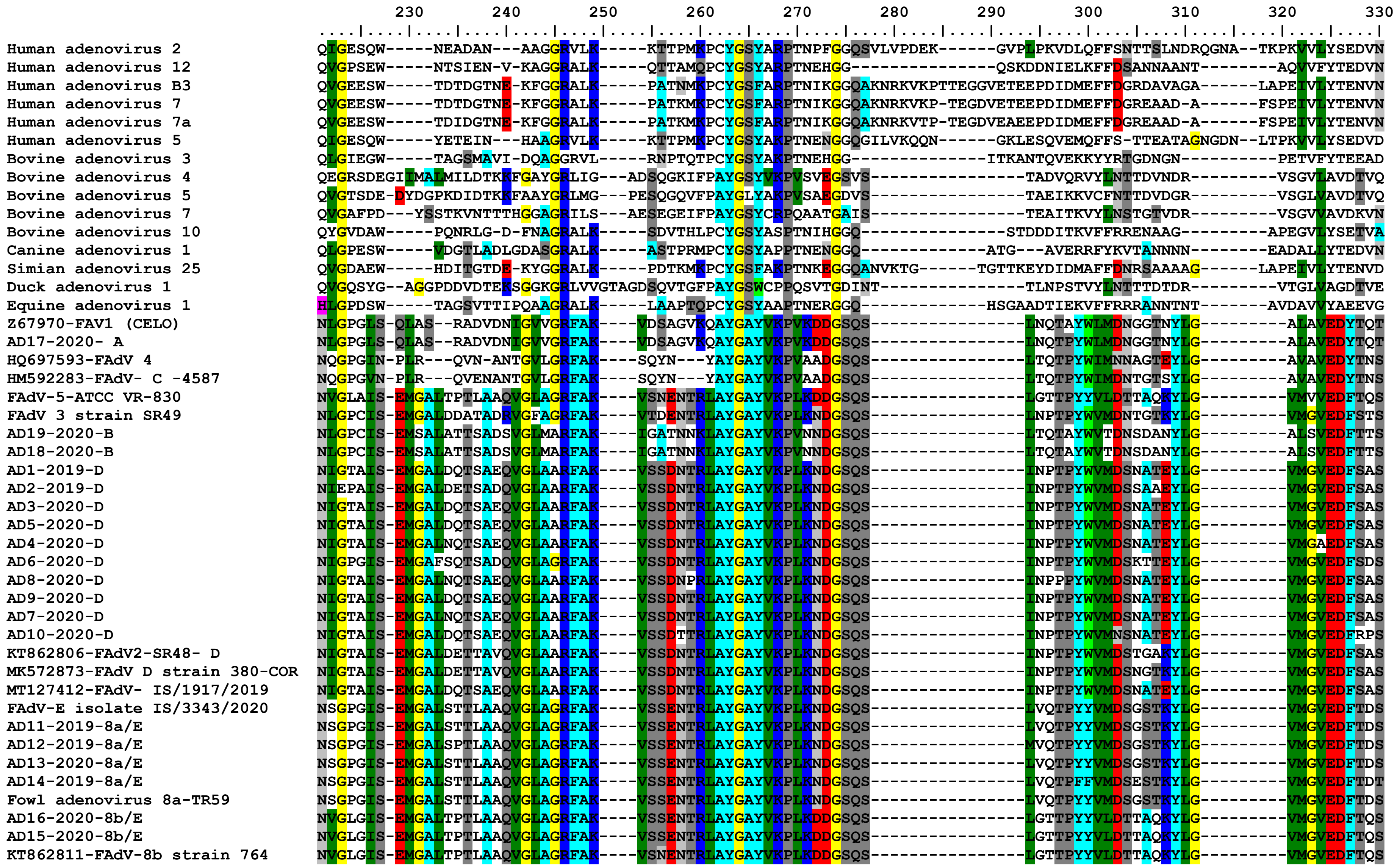
230 240 250 260 270 280 290 300 310 320 330
Human adenovirus 2 QIGESQW-----NEADAN---AAGGRVLK-----KTTPMKPCYGSYARPTNPFGGQSVLVPDEK------GVPLPKVDLQFFSNTTSLNDRQGNA--TKPKVVLYSEDVN
Human adenovirus 12 QVGPSEW-----NTSIEN-V-KAGGRALK-----QTTAMQPCYGSYARPTNEHGG---------------QSKDDNIELKFFDSANNAANT--------AQVVFYTEDVN
Human adenovirus B3 QVGEESW-----TDTDGTNE-KFGGRALK-----PATNMKPCYGSFARPTNIKGGQAKNRKVKPTTEGGVETEEPDIDMEFFDGRDAVAGA------LAPEIVLYTENVN
Human adenovirus 7 QVGEESW-----TDTDGTNE-KFGGRALK-----PATKMKPCYGSFARPTNIKGGQAKNRKVKP-TEGDVETEEPDIDMEFFDGREAAD-A------FSPEIVLYTENVN
Human adenovirus 7a QVGEESW-----TDIDGTNE-KFGGRALK-----PATKMKPCYGSFARPTNIKGGQAKNRKVTP-TEGDVEAEEPDIDMEFFDGREAAD-A------FSPEIVLYTENVN
Human adenovirus 5 QIGESQW-----YETEIN---HAAGRVLK-----KTTPMKPCYGSYAKPTNENGGQGILVKQQN------GKLESQVEMQFFS-TTEATAGNGDN--LTPKVVLYSEDVD
Bovine adenovirus 3 QLGIEGW-----TAGSMAVI-DQAGGRVL-----RNPTQTPCYGSYAKPTNEHGG-------------ITKANTQVEKKYYRTGDNGN---------PETVFYTEEAD
Bovine adenovirus 4 QEGRSDEGIIMALMILDTKKFGAYGRLIG---ADSQGKIFPAYGSYVKPVSVEGSVS---------------TADVQRVYLNTTDVNDR--------VSGVLAVDTVQ
Bovine adenovirus 5 QVGTSDE-DYDGPKDIDTKKFAAYGRLMG---PESQGQVFPAYGLYAKPVSAEGDVS---------------TAEIKKVCFNTTDVDGR--------VSGLVAVDTVQ
Bovine adenovirus 7 QVGAFPD---YSSTKVNTTTHGGAGRILS---AESEGEIFPAYGSYCRPQAATGAIS---------------TEAITKVYLNSTGTVDR--------VSGVVAVDKVN
Bovine adenovirus 10 QYGVDAW-----PQNRLG-D-FNAGRALK-----SDVTHLPCYGSYASPTNIHGGQ---------------STDDDITKVFFRRENAAG--------APEGVLYSETVA
Canine adenovirus 1 QLGPESW-----VDGTLADLGDASGRALK-----ASTPRMPCYGSYAPPTNENGGQ-------------ATG---AVERRFYKVTANNNN--------EADALLYTEDVN
Simian adenovirus 25 QVGDAEW-----HDITGTDE-KYGGRALK-----PDTKMKPCYGSFAKPTNKEGGQANVKTG------TGTTKEYDIDMAFFDNRSAAAAG------LAPEIVLYTENVD
Duck adenovirus 1 QVGQSYG--AGGPDDVDTEKSGGKGRLVVGTAGDSQVTGFPAYGSWCPPQSVTGDINT---------------TLNPSTVYLNTTTDTDR--------VTGLVAGDTVE
Equine adenovirus 1 HLGPDSW-----TAGSVTTIPQAAGRALK-----LAAPTQPCYGSYAAPTNERGGQ-------------HSGAADTIEKVFFRRRANNTNT-------AVDAVVYAEEVG
Z67970-FAV1 (CELO) NLGPGLS-QLAS--RADVDNIGVVGRFAK----VDSAGVKQAYGAYVKPVKDDGSQS-----------------LNQTAYWLMDNGGTNYLG---------ALAVEDYTQT
AD17-2020- A NLGPGLS-QLAS--RADVDNIGVVGRFAK----VDSAGVKQAYGAYVKPVKDDGSQS-----------------LNQTPYWLMDNGGTNYLG---------ALAVEDYTQT
HQ697593-FAdV 4 NQGPGIN-PLR---QVN-ANTGVLGRFAK----SQYN---YAYGAYVKPVAADGSQS-----------------LTQTPYWIMNNAGTEYLG---------AVAVEDYTNS
HM592283-FAdV- C -4587 NQGPGVN-PLR---QVENANTGVLGRFAK----SQYN---YAYGAYVKPVAADGSQS-----------------LTQTPYWIMDNTGTSYLG---------AVAVEDYTNS
FAdV-5-ATCC VR-830 NVGLAIS-EMGALTPTLAAQVGLAGRFAK----VSNENTRLAYGAYVKPLKDDGSQS-----------------LGTTPYYVLDTTAQKYLG---------VMVVEDFTQS
FAdV 3 strain SR49 NLGPCIS-EMGALDDATADRVGFAGRFAK----VTDENTRLAYGAYVKPLKNDGSQS-----------------LNPTPYWVMDNTGTKYLG---------VMGVEDFSTS
AD19-2020-B NLGPCIS-EMSALATTSADSVGLMARFAK----IGATNNKLAYGAYVKPVNNDGSQS-----------------LTQTAYWVTDNSDANYLG---------ALSVEDFTTS
AD18-2020-B NLGPCIS-EMSALATTSADSVGLMARFAK----IGATNNKLAYGAYVKPVNNDGSQS-----------------LTQTAYWVTDNSDANYLG---------ALSVEDFTTS
AD1-2019-D NIGTAIS-EMGALDQTSAEQVGLAARFAK----VSSDNTRLAYGAYVKPLKNDGSQS-----------------INPTPYWVMDSNATEYLG---------VMGVEDFSAS
AD2-2019-D NIEPAIS-EMGALDETSADQVGLAARFAK----VSSDNTRLAYGAYVKPLKNDGSQS-----------------INPTPYWVMDSSAAEYLG---------VMGVEDFSAS
AD3-2020-D NIGTAIS-EMGALDQTSAEQVGLAARFAK----VSSDNTRLAYGAYVKPLKNDGSQS-----------------INPTPYWVMDSNATEYLG---------VMGVEDFSAS
AD5-2020-D NIGTAIS-EMGALDQTSAEQVGLAARFAK----VSSDNTRLAYGAYVKPLKNDGSQS-----------------INPTPYWVMDSNATEYLG---------VMGVEDFSAS
AD4-2020-D NIGTAIS-EMGALNQTSAEQVGLAARFAK----VSSDNTRLAYGAYVKPLKNDGSQS-----------------INPTPYWVMDSNATEYLG---------VMGAEDFSAS
AD6-2020-D NIGPGIS-EMGAFSQTSADQVGLAGRFAK----VSSDNTRLAYGAYVKPLKNDGSQS-----------------INPTPYWVMDSKTTEYLG---------VMGVEDFSDS
AD8-2020-D NIGTAIS-EMGALNQTSAEQVGLAARFAK----VSSDNPRLAYGAYVKPLKNDGSQS-----------------INPPPYWVMDSNATEYLG---------VMGVEDFSAS
AD9-2020-D NIGTAIS-EMGALDQTSAEQVGLAARFAK----VSSDNTRLAYGAYVKPLKNDGSQS-----------------INPTPYWVMDSNATEYLG---------VMGVEDFSAS
AD7-2020-D NIGTAIS-EMGALNQTSAEQVGLAARFAK----VSSDNTRLAYGAYVKPLKNDGSQS-----------------INPTPYWVMDSNATEYLG---------VMGVEDFSAS
AD10-2020-D NIGTAIS-EMGALDQTSAEQVGLAARFAK----VSSDTTRLAYGAYVKPLKNDGSQS-----------------INPTPYWVMNSNATEYLG---------VMGVEDFRPS
KT862806-FAdV2-SR48- D NIGTAIS-EMGALDETTAVQVGLAARFAK----VSSDNTRLAYGAYVKPLKNDGSQS-----------------INPTPYWVMDSTGAKYLG---------VMGVEDFSAS
MK572873-FAdV D strain 380-COR NIGTAIS-EMGALDETTAVQVGLAARFAK----VSSDNTRLAYGAYVKPLKNDGSQS-----------------INPTPYWVMDSNGTKYLG---------VMGVEDFSAS
MT127412-FAdV- IS/1917/2019 NIGTAIS-EMGALDQTSAEQVGLAARFAK----VSSDNTRLAYGAYVKPLKNDGSQS-----------------INPTPYWVMDSNATEYLG---------VMGVEDFSAS
FAdV-E isolate IS/3343/2020 NSGPGIS-EMGALSTTLAAQVGLAGRFAK----VSSENTRLAYGAYVKPLKNDGSQS-----------------LVQTPYYVMDSGSTKYLG---------VMGVEDFTDS
AD11-2019-8a/E NSGPGIS-EMGALSTTLAAQVGLAGRFAK----VSSENTRLAYGAYVKPLKNDGSQS-----------------VVQTPYYVMDSGSTKYLG---------VMGVEDFTDS
AD12-2019-8a/E NSGPGIS-EMGALSPTLAAQVGLAGRFAK----VSSENTRLAYGAYVKPLKNDGSQS-----------------MVQTPYYVMDSGSTKYLG---------VMGVEDFTDS
AD13-2020-8a/E NSGPGIS-EMGALSTTLAAQVGLAGRFAK----VSSENTRLAYGAYVKPLKNDGSQS-----------------IVQTPYYVMDSGSTKYLG---------VMGVEDFTDS
AD14-2019-8a/E NSGPGIS-EMGALSTTLAAQVGLAGRFAK----VSSENTRLAYGAYVKPLKNDGSQS-----------------LVQTPFFVMDSESTKYLG---------VMGVEDFTDT
Fowl adenovirus 8a-TR59 NSGPGIS-EMGALSTTLAAQVGLAGRFAK----VSSENTRLAYGAYVKPLKNDGSQS-----------------LVQTPYYVMDSGSTKYLG---------VMGVEDFTDS
AD16-2020-8b/E NVGLGIS-EMGALTPTLAAQVGLAGRFAK----VSSENTRLAYGAYVKPLKDDGSQS-----------------LGTTPYYVLDTTAQKYLG---------VMGVEDFTQS
AD15-2020-8b/E NVGLGIS-EMGALTPTLAAQVGLAGRFAK----VSSENTRLAYGAYVKPLKDDGSQS-----------------LGTTPYYVLDTTAQKYLG---------VMGVEDFTQS
KT862811-FAdV-8b strain 764 NVGLGIS-EMGALTPTLAAQVGLAGRFAK----VSNENTRLAYGAYVKPLKDDGSQS-----------------LGTTPYYVLDTTAQKYLG---------VMGVEDFTQS

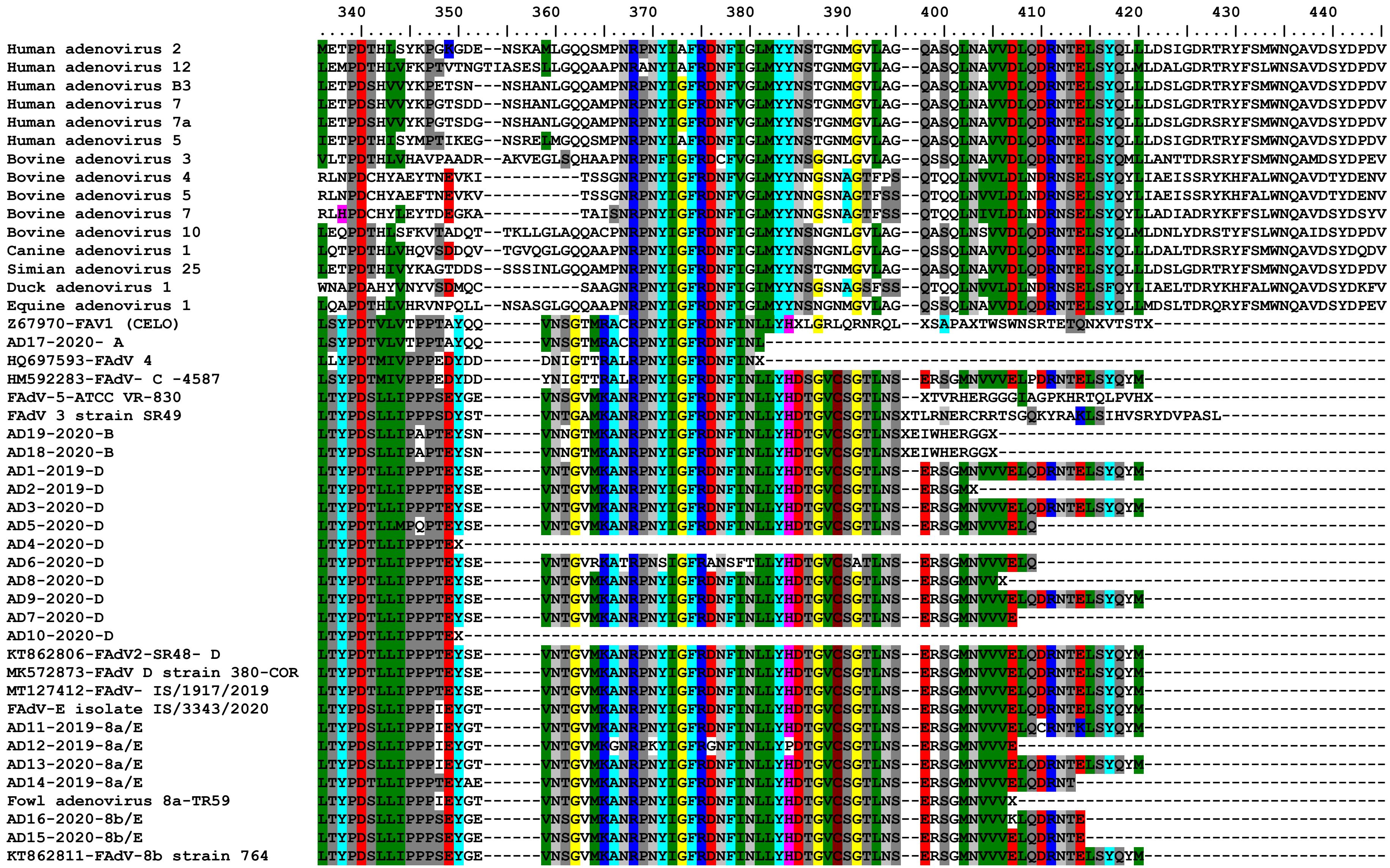

340 350 360 370 380 390 400 410 420 430 440
Human adenovirus 2 METPDTHLSYKPGKGDE--NSKAMLGQQSMPNRPNYIAFRDNFIGLMYYNSTGNMGVLAG--QASQLNAVVDLQDRNTELSYQLLLDSIGDRTRYFSMWNQAVDSYDPDV
Human adenovirus 12 LEMPDTHLVFKPTVTNGTIASESLLGQQAAPNRANYIAFRDNFIGLMYYNSTGNMGVLAG--QASQLNAVVDLQDRNTELSYQLMLDALGDRTRYFSLWNSAVDSYDPDV
Human adenovirus B3 LETPDSHVVYKPETSN---NSHANLGQQAMPNRPNYIGFRDNFVGLMYYNSTGNMGVLAG--QASQLNAVVDLQDRNTELSYQLLLDSLGDRTRYFSMWNQAVDSYDPDV
Human adenovirus 7 LETPDSHVVYKPGTSDD--NSHANLGQQAMPNRPNYIGFRDNFVGLMYYNSTGNMGVLAG--QASQLNAVVDLQDRNTELSYQLLLDSLGDRTRYFSMWNQAVDSYDPDV
Human adenovirus 7a LETPDSHVVYKPGTSDG--NSHANLGQQAMPNRPNYIGFRDNFVGLMYYNSTGNMGVLAG--QASQLNAVVDLQDRNTELSYQLLLDSLGDRSRYFSMWNQAVDSYDPDV
Human adenovirus 5 IETPDTHISYMPTIKEG--NSRELMGQQSMPNRPNYIAFRDNFIGLMYYNSTGNMGVLAG--QASQLNAVVDLQDRNTELSYQLLLDSIGDRTRYFSMWNQAVDSYDPDV
Bovine adenovirus 3 VLTPDTHLVHAVPAADR--AKVEGLSQHAAPNRPNFIGFRDCFVGLMYYNSGGNLGVLAG--QSSQLNAVVDLQDRNTELSYQMLLANTTDRSRYFSMWNQAMDSYDPEV
Bovine adenovirus 4 RLNPDCHYAEYTNEVKI----------TSSGNRPNYIGFRDNFVGLMYYNNGSNAGTFPS--QTQQLNVVLDLNDRNSELSYQYLIAEISSRYKHFALWNQAVDTYDENV
Bovine adenovirus 5 RLNPDCHYAEFTNEVKV----------TSSGNRPNYIGFRDNFVGLMYYNNGSNAGTFSS--QTQQLNVVLDLNDRNSELSYQYLIAEISSRYKHFALWNQAVDTYDENV
Bovine adenovirus 7 RLHPDCHYLEYTDEGKA----------TAISNRPNYIGFRDNFIGLMYYNNGSNAGTFSS--QTQQLNIVLDLNDRNSELSYQYLLADIADRYKFFSLWNQAVDSYDSYV
Bovine adenovirus 10 LEQPDTHLSFKVTADQT--TKLLGLAQQACPNRPNYIGFRDNFIGLMYYNSNGNLGVLAG--QASQLNSVVDLQDRNTELSYQLMLDNLYDRSTYFSLWNQAIDSYDPDV
Canine adenovirus 1 LQTPDTHLVHQVSDDQV--TGVQGLGQQAAPNRPNYIGFRDNFIGLMYYNSNGNLGVLAG--QSSQLNAVVDLQDRNTELSYQLLLDALTDRSRYFSMWNQAVDSYDQDV
Simian adenovirus 25 LETPDTHIVYKAGTDDS--SSSINLGQQAMPNRPNYIGFRDNFIGLMYYNSTGNMGVLAG--QASQLNAVVDLQDRNTELSYQLLLDSLGDRTRYFSMWNQAVDSYDPDV
Duck adenovirus 1 WNAPDAHYVNYVSDMQC----------SAAGNRPNYIGFRDNFIGIMYYNSGSNAGSFSS--QTQQLNVVLDLNDRNSELSFQYLIAELTDRYKHFALWNQAVDSYDKFV
Equine adenovirus 1 LQAPDTHLVHRVNPQLL--NSASGLGQQAAPNRPNYIGFRDNFIGLMYYNSNGNMGVLAG--QSSQLNAVVDLQDRNTELSYQLLMDSLTDRQRYFSMWNQAVDSYDPEV
Z67970-FAV1 (CELO) LSYPDTVLVTPPTAYQQ------VNSGTMRACRPNYIGFRDNFINLLYHXLGRLQRNRQL--XSAPAXTWSWNSRTETQNXVTSTX------------------------
AD17-2020- A LSYPDTVLVTPPTAYQQ------VNSGTMRACRPNYIGFRDNFINL---
HQ697593-FAdV 4 LLYPDTMIVPPPEDYDD------DNIGTTRALRPNYIGFRDNFINX---
HM592283-FAdV- C -4587 LSYPDTMIVPPPEDYDD------YNIGTTRALRPNYIGFRDNFINLLYHDSGVCSGTLNS--ERSGMNVVVELPDRNTELSYQYM---------------------------
FAdV-5-ATCC VR-830 LTYPDSLLIPPPSEYGE------VNSGVMKANRPNYIGFRDNFINLLYHDTGVCSGTLNS--XTVRHERGGGIAGPKHRTQLPVHX--------------------------
FAdV 3 strain SR49 LTYPDSLLIPPPSDYST------VNTGAMKANRPNYIGFRDNFINLLYHDTGVCSGTLNSXTLRNERCRRTSGQKYRAKLSIHVSRYDVPASL-------------------
AD19-2020-B LTYPDSLLIPAPTEYSN------VNNGTMKANRPNYIGFRDNFINLLYHDTGVCSGTLNSXEIWHERGGX--
AD18-2020-B LTYPDSLLIPAPTEYSN------VNNGTMKANRPNYIGFRDNFINLLYHDTGVCSGTLNSXEIWHERGGX--
AD1-2019-D LTYPDTLLIPPPTEYSE------VNTGVMKANRPNYIGFRDNFINLLYHDTGVCSGTLNS--ERSGMNVVVELQDRNTELSYQYM---------------------------
AD2-2019-D LTYPDTLLIPPPTEYSE------VNTGVMKANRPNYIGFRDNFINLLYHDTGVCSGTLNS--ERSGMX--
AD3-2020-D LTYPDTLLIPPPTEYSE------VNTGVMKANRPNYIGFRDNFINLLYHDTGVCSGTLNS--ERSGMNVVVELQDRNTELSYQYM---------------------------
AD5-2020-D LTYPDTLLMPQPTEYSE------VNTGVMKANRPNYIGFRDNFINLLYHDTGVCSGTLNS--ERSGMNVVVELQ--------------------------------------
AD4-2020-D LTYPDTLLIPPPTEX---
AD6-2020-D LTYPDTLLIPPPTEYSE------VNTGVRKATRPNSIGFRANSFTLLYHDTGVCSATLNS--ERSGMNVVVELQ--------------------------------------
AD8-2020-D LTYPDTLLIPPPTEYSE------VNTGVMKANRPNYIGFRDNFINLLYHDTGVCSGTLNS--ERSGMNVVX---
AD9-2020-D LTYPDTLLIPPPTEYSE------VNTGVMKANRPNYIGFRDNFINLLYHDTGVCSGTLNS--ERSGMNVVVELQDRNTELSYQYM---------------------------
AD7-2020-D LTYPDTLLIPPPTEYSE------VNTGVMKANRPNYIGFRDNFINLLYHDTGVCSGTLNS--ERSGMNVVVE--
AD10-2020-D LTYPDTLLIPPPTEX---
KT862806-FAdV2-SR48- D LTYPDTLLIPPPTEYSE------VNTGVMKANRPNYIGFRDNFINLLYHDTGVCSGTLNS--ERSGMNVVVELQDRNTELSYQYM---------------------------
MK572873-FAdV D strain 380-COR LTYPDTLLIPPPTEYSE------VNTGVMKANRPNYIGFRDNFINLLYHDTGVCSGTLNS--ERSGMNVVVELQDRNTELSYQYM---------------------------
MT127412-FAdV- IS/1917/2019 LTYPDTLLIPPPTEYSE------VNTGVMKANRPNYIGFRDNFINLLYHDTGVCSGTLNS--ERSGMNVVVELQDRNTELSYQYM---------------------------
FAdV-E isolate IS/3343/2020 LTYPDSLLIPPPIEYGT------VNTGVMKANRPNYIGFRDNFINLLYHDTGVCSGTLNS--ERSGMNVVVELQDRNTELSYQYM---------------------------
AD11-2019-8a/E LTYPDSLLIPPPIEYGT------VNTGVMKANRPNYIGFRDNFINLLYHDTGVCSGTLNS--ERSGMNVVVELQCRNTKLSYQYM---------------------------
AD12-2019-8a/E LTYPDSLLIPPPIEYGT------VNTGVMKGNRPKYIGFRGNFINLLYPDTGVCSGTLNS--ERSGMNVVVE--
AD13-2020-8a/E LTYPDSLLIPPPIEYGT------VNTGVMKANRPNYIGFRDNFINLLYHDTGVCSGTLNS--ERSGMNVVVELQDRNTELSYQYM---------------------------
AD14-2019-8a/E LTYPDTLLIPPPTEYAE------VNTGVMKANRPNYIGFRDNFINLLYHDTGVCSGTLNS--ERSGMNVVVELQDRNT----------------------------------
Fowl adenovirus 8a-TR59 LTYPDSLLIPPPIEYGT------VNTGVMKANRPNYIGFRDNFINLLYHDTGVCSGTLNS--ERSGMNVVVX--
AD16-2020-8b/E LTYPDSLLIPPPSEYGE------VNSGVMKANRPNYIGFRDNFINLLYHDTGVCSGTLNS--ERSGMNVVVKLQDRNTE---------------------------------
AD15-2020-8b/E LTYPDSLLIPPPSEYGE------VNSGVMKANRPNYIGFRDNFINLLYHDTGVCSGTLNS--ERSGMNVVVELQDRNTE---------------------------------
KT862811-FAdV-8b strain 764 LTYPDSLLIPPPSEYGE------VNSGVMKANRPNYIGFRDNFINLLYHDTGVCSGTLNS--ERSGMNVVVELQDRNTELSYQYM---------------------------
